# Autophagy, Oxidative Stress, and Alcoholic Liver Disease: A Systematic Review and Potential Clinical Applications

**DOI:** 10.3390/antiox12071425

**Published:** 2023-07-14

**Authors:** Daniel Salete-Granado, Cristina Carbonell, David Puertas-Miranda, Víctor-José Vega-Rodríguez, Marina García-Macia, Ana Belén Herrero, Miguel Marcos

**Affiliations:** 1 Instituto de Investigación Biomédica de Salamanca (IBSAL), 37007 Salamanca, Spain; dsaleteg@usal.es (D.S.-G.); carbonell@usal.es (C.C.); dpuertas@saludcastillayleon.es (D.P.-M.); victorjvega@saludcastillayleon.es (V.-J.V.-R.); marinagarciamacia@usal.es (M.G.-M.); anah@usal.es (A.B.H.); 2 Hospital Universitario de Salamanca, 37007 Salamanca, Spain; 3 Unidad de Medicina Molecular, Departamento de Medicina, Universidad de Salamanca, 37007 Salamanca, Spain; 4 Instituto de Biología Funcional y Genómica (IBFG), Universidad de Salamanca, 37007 Salamanca, Spain

**Keywords:** autophagy, oxidative stress, alcoholic liver disease, alcohol, ethanol, macroautophagy, mitophagy, redox, antioxidant, rapamycin

## Abstract

Ethanol consumption triggers oxidative stress by generating reactive oxygen species (ROS) through its metabolites. This process leads to steatosis and liver inflammation, which are critical for the development of alcoholic liver disease (ALD). Autophagy is a regulated dynamic process that sequesters damaged and excess cytoplasmic organelles for lysosomal degradation and may counteract the harmful effects of ROS-induced oxidative stress. These effects include hepatotoxicity, mitochondrial damage, steatosis, endoplasmic reticulum stress, inflammation, and iron overload. In liver diseases, particularly ALD, macroautophagy has been implicated as a protective mechanism in hepatocytes, although it does not appear to play the same role in stellate cells. Beyond the liver, autophagy may also mitigate the harmful effects of alcohol on other organs, thereby providing an additional layer of protection against ALD. This protective potential is further supported by studies showing that drugs that interact with autophagy, such as rapamycin, can prevent ALD development in animal models. This systematic review presents a comprehensive analysis of the literature, focusing on the role of autophagy in oxidative stress regulation, its involvement in organ–organ crosstalk relevant to ALD, and the potential of autophagy-targeting therapeutic strategies.

## 1. Introduction

Alcohol consumption causes multi-organ damage and is linked to a wide variety of diseases. In 2016, ethanol intake caused an estimated 3 million alcohol-related deaths and resulted in 132.6 million alcohol-related disability-adjusted life years worldwide [1]. Alcohol intake causes a wide spectrum of damage in the liver, ranging from steatosis to alcoholic hepatitis, cirrhosis, and hepatocellular carcinoma [2,3,4]. One-quarter of cirrhosis deaths and one-fifth of liver cancer deaths worldwide in 2019 were associated with alcohol toxicity [4]. Some of the mechanisms underlying this toxicity are: (1) ethanol metabolism due to acetaldehyde accumulation, (2) an increase in the nicotinamide adenine dinucleotide (NAD)H/NAD^+^ ratio, and/or (3) ROS generation [5]. Oxidative stress may be involved in functional and structural changes in mitochondria, leading to impaired oxidative phosphorylation, increased mitochondrial DNA (mtDNA) damage, and changes in mitochondrial protein profiles [6,7,8]. An increase in ROS levels leads to lipid peroxidation, as lipids accumulate in the liver during ethanol metabolism, which can escalate organ damage [9,10].

Simultaneously, oxidative stress can induce autophagy via different pathways. Autophagy is a dynamic, evolutionarily conserved process that aims to maintain cellular homeostasis, in which defective organelles, toxic proteins, and various other aggregates on the cytoplasmic surface are degraded and recycled in the effort to promote cell survival [11]. Ethanol-induced autophagy in the liver does not appear to be related to long-lived proteins; however, it does seem to be selective for damaged mitochondria and accumulated lipid droplets [12,13,14]. The stimulation and suppression of autophagy have been found to alleviate and exacerbate acute and chronic alcohol-related liver damage, respectively [15,16,17], paving the way for potential treatment development. In addition to affecting autophagic processes and oxidative stress in the liver, alcohol can dysregulate autophagy in several other organs, including the gut [18,19], adipose tissue [20], pancreas [21], brain [22], skeletal muscle [23], and heart [24]. Thus, organ–organ crosstalk—defined as complex, mutual, signaling factor-mediated communication between distant organs [25] such as the liver and the gut [26], adipose tissue [27,28], and brain [29]—has received increasing research interest. In this review, we summarize the current evidence available regarding the involvement of autophagic pathways in alcoholic liver disease (ALD) and their relationship with oxidative stress, as well as organ–organ crosstalk and treatment options ALD.

## 2. Materials and Methods

### 2.1. Search Strategy

We conducted a systematic review of publications on the mechanisms of oxidative stress and autophagy, as well as organ–organ crosstalk and related therapeutic strategies, in the context of ALD. Two investigators (CC and DS) independently performed the bibliographic search, and any divergence of opinion was resolved by consensus with the senior author (MM). We searched the PubMed, Web of Science, Scopus, and Embase electronic databases for publications up until 15 November 2022 using the following search terms alone and in combination as medical subject headings: “autophagy” or “p62” or “SQSTM1” or “autophagy-lysosome system” and “oxidative stress” or “redox” or “reactive oxygen species” or “cellular stress” or “antioxidants” and “alcoholic liver disease” or “alcoholic diseases” or “alcohol” or “ethanol”. No language restriction was applied to the initial search, although only the abstracts of articles published in languages other than English were considered. We manually scanned the reference lists of the retrieved publications to identify additional relevant articles. In addition, the search was supplemented by using the Medline “related articles” option and consulting review articles on the topic. Abstracts in meeting proceedings were not evaluated. Rayyan software (https://www.rayyan.ai/, accessed on 13 January 2023) was used to manage citations and identify and remove duplicates [30]. The protocol for the systematic review was registered with PROSPERO (ID: CRD42023435089).

### 2.2. Article Selection and Data Extraction

Published articles offering information on the mechanisms of oxidative stress and autophagy pathways in the presence of alcohol were selected. Studies with human samples, cell lines, and animal models were considered. Studies that did not directly evaluate the effects of alcohol, including autophagy, were excluded. Data recorded from each article included methodological details (cell type, murine model, or pharmacological drug used) and the molecular effects on autophagy and oxidative stress; these data were extracted independently by two authors for each dataset (DS, DPM, CC, and VJVR). The literature was summarized in tables, where appropriate.

## 3. Results and Literature Review

### 3.1. Studies Selected

**Figure 1:**
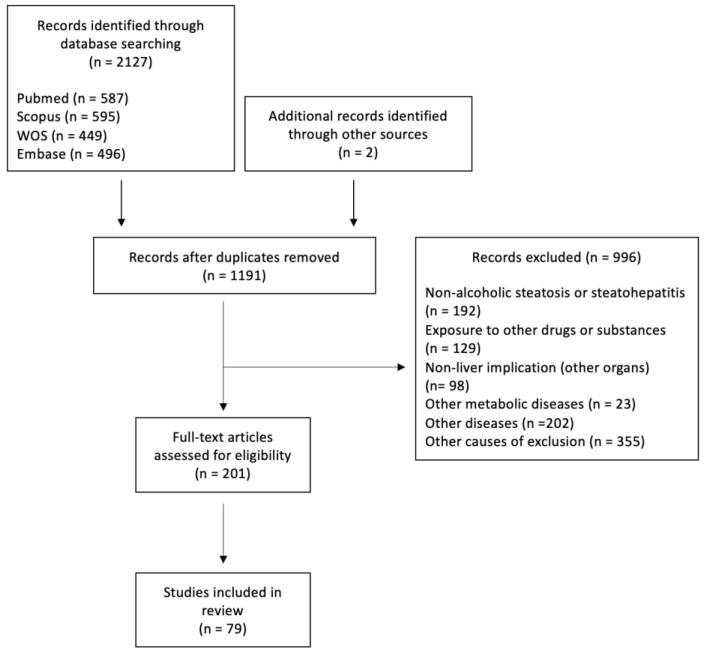
Flow of study selection.

Our initial search yielded 2127 articles, of which 996 were excluded and 200 were assessed for eligibility (Figure 1). In total, 78 articles were included in this systematic review. The literature regarding the roles of autophagy in hepatocytes and liver lysates, as well as in macrophages and hepatic stellate cells (HSCs) under both chronic and acute ethanol intake conditions, is comprehensively summarized in Table 1, Table 2, Table 3, Table 4 and Table 5. Acute ethanol models were defined as the exposure of cells for 24 h or less or intake of ethanol on one occasion or for less than 24 h. Studies focusing on the effects of specific therapeutic agents rather than describing the novel mechanisms involved in ethanol-induced autophagy are summarized in Table 6.

### 3.2. Ethanol, ALD, and Oxidative Stress

#### 3.2.1. Ethanol Metabolism

Hepatocytes remove ethanol from the bloodstream via non-oxidative and oxidative metabolism. Ethanol is metabolized oxidatively via two major pathways: alcohol dehydrogenase (ADH) and cytochrome P450 enzymes (in this case, CYP2E1) [108]. These pathways produce ethanol metabolites, such as acetaldehyde and ROS, and deplete the stores of some antioxidants such as glutathione (GSH) [109,110]. The metabolites produced induce oxidative stress [including endoplasmic reticulum (ER) stress and mitochondrial damage], autophagy, and inflammation, which add to the hepatic inflammation caused by the action of bacterial lipopolysaccharide (LPS) [111,112,113,114].

The main alcohol metabolism pathway is initiated by ADH [113], an NAD^+^-requiring enzyme that is strongly expressed in hepatocytes and oxidizes ethanol to acetaldehyde in the cytosol [108], decreasing the NAD^+^/NADH ratio [115]. In normal liver, acetaldehyde is quickly metabolized to acetate by aldehyde dehydrogenase (ALDH). In chronic alcohol users, the ADH/ALDH pathway becomes saturated and generates reactive aldehydes and lipid hydroperoxides that can bind to DNA and proteins, producing adducts that further induce hepatocyte damage and inflammation [116,117]. The decrease in the NAD^+^/NADH ratio also promotes fat accumulation in the liver by reducing fatty acid oxidation (through peroxisome proliferator–activated receptor-α target genes) and enhancing fatty acid synthesis (by increasing the expression levels of lipogenic genes such as SREBP1c) [118,119,120], reducing sirtuin-1 (SIRT1)-related autophagy by decreasing transcription factor EB (TFEB) deacetylation [121], and increasing oxidative stress, as the re-oxidation of NADH to NAD^+^ in mitochondria requires ROS production [122].

The second major pathway centers around CYP2E1, a nicotinamide adenine dinucleotide phosphate (NADPH)-requiring enzyme, and gains relevance over the ADH pathway with chronic ethanol intake [113]. CYP2E1 metabolizes ethanol to acetaldehyde by converting NADPH and oxygen to NADP^+^ and water, resulting in the generation of ROS such as H_2_O_2_, hydroxyl (HO^•−^), and carbon-centered HO^•−^ [123,124]. Through this process, CYP2E1 facilitates DNA and protein adduct formation, activates stress proteins, induces ER stress, and affects lysosomal function and autophagy, leading to mitochondrial damage, hepatocellular death, and hepatic carcinogenesis via oxidative DNA damage [113,125].

Nicotinamide N-methyltransferase (NNMT) is another important enzyme involved in liver metabolism and alcoholic liver disease [126,127]. NNMT is highly expressed in the human liver and plays a crucial role in maintaining NAD+ homeostasis [128]. It catalyzes the methylation of nicotinamide and similar compounds using the methyl donor S-adenosyl methionine (SAM-e) to produce S-adenosyl-L-homocysteine (SAH) and 1-methylnicotinamide. NNMT can affect the autophagic pathway [129,130] and may counteract oxidative stress in liver vessels due to its putative protective role in the endothelium [131]. Increased NNMT in the liver is associated with a better metabolic profile, including reduced serum triglyceride and free fatty acid levels [132]. In the liver, 1-methylnicotinamide produced by the NNMT degradation of nicotinamide increases sirtuin 1 (SIRT1) by inhibiting its degradation [132].

Thus, ROS generation through alcohol metabolism, with a reduction in the NAD^+^/NADH ratio and the activation of CYP2E1, is a major hallmark of alcohol-related oxidative stress. One of the best indicators of ROS overproduction is an increase in hepatic CYP2E1 levels. ROS-induced oxidative stress is closely related to protein modification, lipid peroxidation, mitochondrial damage, inflammation, iron overload, and antioxidant responses.

#### 3.2.2. Protein Modifications

ROS induce reversible protein modifications, mainly at the level of the sulfur-containing residues cysteine and methionine, indicating a redox-based signal. Oxidative stress can cause changes in protein structure, localization, physical interactions, and post-translational modifications through the oxidative modification of reactive cysteines [133]. These changes can affect the immune response against neoantigens [134]. In liver sinusoidal endothelial cells, oxidative stress can also alter the proper functioning of fenestrae through spectrin oxidation [135]. For instance, oxidative stress reduces disulfide bond formation and causes the accumulation of unfolded proteins, triggering additional ER stress [136]. Cells counteract these protein modifications via three main signaling pathways: inositol-requiring transmembrane kinase/endoribonuclease 1 (IRE1), activating transcription factor 6, and protein kinase RNA-like ER kinase. Collectively, they form the unfolded protein response (UPR), which occurs mainly via the ubiquitin–proteasome pathway [15].

Alcohol consumption also causes the synthesis of reactive nitrogen species (RNS) in the liver [137]. The RNS NO causes S-nitrosylation in proteins [e.g., c-Jun N-terminal kinase 1 (JNK1) and inhibitor κB kinase β] and lysosomal enzymes (i.e., hexosaminidase B and cathepsin B), which impair hepatic autophagy, especially mitophagy (mitochondrial macroautophagy) [138,139].

#### 3.2.3. Lipid Peroxidation and DNA Alteration

ROS can react with lipid species to promote lipid peroxidation, inducing apoptosis and ferroptosis [9,140], among other changes. Lipid peroxidation products can bind to DNA and enhance carcinogenesis by producing etheno-DNA adducts and mutations in oncogenes and onco-suppressor genes [10,141,142]. These adducts have been identified in the livers of patients with ALD [143]. In addition, ROS can directly modify DNA, thereby affecting cell viability. HO^•−^ can directly attack the DNA backbone, mainly guanine, due to its low redox potential. The main products of its oxidation are 8-hydroxyguanine and 8-hydroxydeoxyguanosine, which are mutagenic and carcinogenic [144].

#### 3.2.4. Mitochondrial Damage

ALD is characterized by structural and functional abnormalities in hepatic mitochondria, including enlargement [145], mtDNA damage [6], reduced adenosine triphosphate (ATP) levels [146], mitochondrial protein synthesis [147], increased ROS production [7,8], and alterations in mitochondrial membrane permeability and mitochondrial permeability transition, resulting in intrinsic and extrinsic apoptosis [124]. In intrinsic apoptosis, cellular death occurs via the release of cytochrome C and other pro-apoptotic factors that interact with apoptotic protease activating factor 1 and caspase-9 [148]. In the extrinsic pathway, cell death is triggered by the ROS-induced release of apoptosis signal-regulating kinase 1, a member of the mitogen-activated protein kinase (MAPK) family, resulting in the cleavage of pro-caspase-3 to active caspase-3 [149,150]. Mitochondrial permeability transition has been found to activate caspase-3 in hepatocytes in a p38 MAPK–dependent manner [151].

ROS also harm mtDNA integrity by affecting mtDNA-coded proteins and RNA transcription, which in turn regulates the mitochondrial respiratory chain. A vicious cycle is established in which mitochondria with oxidized mtDNA and limited repair mechanisms [152] become dysfunctional and produce abundant ROS, leading to further mitochondrial impairment. This loop can ultimately result in severe nuclear DNA damage and cell death [153]. Mitophagy degrades mitochondria with damaged DNA [154] and enhances longevity in rodent models [155]. However, the autophagy rate declines with age and chronic alcohol intake [53], promoting the accumulation of mtDNA mutations and a decline in mitochondrial function [156].

#### 3.2.5. Inflammation

ROS also play a key role in the development of ethanol-induced inflammation. The depletion of mitochondrial GSH by CYP2E1 activation [157,158] is one factor that links inflammation with oxidative stress as it impairs hepatocyte tolerance to tumor necrosis factor-α (TNF-α) [115]. TNF-α exacerbates oxidative damage and inflammation and stimulates MAPK activation [159], resulting in ROS accumulation through superoxide (O_2_^•−^) generation. This process causes oxidative damage and eventual TNF-α production, perpetuating the cycle [150,160]. The overexpression of CYP2E1 due to ethanol-induced oxidative stress also induces inflammation via the Notch1 pathway [161], and ROS mediate interleukin (IL)-1β and IL-18 signaling via inflammasome NLRP3 activation [162,163].

Nicotinamide adenine dinucleotide phosphate oxidases (NOXs) are also important sources of inflammation-related ROS generation, as they generate O_2_^•−^ from oxygen using NAD(P)H. In mice, chronic alcohol consumption increased NOX4 expression in the mitochondrial fraction and NOX4 inhibition ameliorated alcohol-induced liver damage [164]. Upon ethanol administration, NOX-derived ROS are key mediators of nuclear factor-κB activation and subsequent TNF-α production [165], sensitizing Kupffer cells to LPS, thereby contributing to ALD [166].

#### 3.2.6. Iron Overload

Metals such as zinc and iron are involved in ROS-induced oxidative stress caused by ethanol intake. Hepatic iron overload has been observed in approximately 50% of patients with ALD [167]. It causes cellular damage and ferroptosis, which may contribute to ROS-associated alcohol toxicity through Fenton reactions [168] and cause lipid peroxidation and subsequent cell membrane damage and rupture, thereby promoting the autophagy-dependent release of damage-associated molecular patterns [169].

#### 3.2.7. Protective Mechanisms and the Antioxidant Response

Several mechanisms, including the involvement of antioxidants, counteract the harmful effects of ethanol-induced oxidative stress. The antioxidant enzymes superoxide dismutase (SOD), catalases, and GSH peroxidases, in concert with other proteins, are responsible for ROS removal and restoring the reduced protein and lipid pool [170]. Ethanol inhibits the expression of antioxidant enzymes (e.g., SOD1) and depletes the levels of non-enzyme antioxidants (e.g., GSH), thereby reducing the cellular ability to modulate oxidative stress [171,172]. ROS weaken this antioxidant response by S-glutathionylation, which reduces the expression of downstream antioxidants, such as SOD2, catalases, and sestrin 3 via the phosphoinositide 3 kinase (PI3K)/AKT pathway [173]. This process also increases mitochondrial generation of H_2_O_2_, facilitating further oxidative damage [174].

### 3.3. Autophagy and ALD

The three main types of autophagy which have different means of cargo delivery to the lysosome are macroautophagy, microautophagy, and chaperone-mediated autophagy [15,175]. In macroautophagy, cytosolic materials are sequestered by autophagosomes and then fused with lysosomes for degradation. In microautophagy, cytoplasmic cargo is engulfed directly into the lysosomes [176]. Chaperone-mediated autophagy involves the direct shuttling of specific proteins across the lysosomal membrane for lumen degradation [177].

Macroautophagy can be classified according to the type of organelle or structure that is selectively degraded as mitophagy (mitochondria), lipophagy (lipid droplets) [178,179,180], ribophagy (ribosomes), ER-phagy (ER), and pexophagy (peroxisomes) [181], among others. This selective form of autophagy is made possible by adaptor proteins such as p62 and specific receptors [182].

#### 3.3.1. The Macroautophagy Pathway

Several multi-molecular complexes contribute to autophagosome formation in macroautophagy: (1) the uncoordinated-51-like kinase (ULK) and Beclin-1 complexes, (2) the antithymocyte globulin (Atg)9–Atg2–Atg18 complex, and (3) the Atg5–Atg12–Atg16 and Atg7–Atg3–Atg8/microtubule-associated protein light chain 3 (LC3) conjugation systems [15,16,17]. The initiation of the macroautophagic pathway involves assembly-site phagophore formation, which is regulated by the ULK complex (composed of ULK), the focal adhesion kinase family-interacting protein of 200 kDa, and Atg13 [183]. This complex is activated by adenosine monophosphate-activated protein kinase (AMPK) and inhibited by the mammalian target of rapamycin complex 1 (mTORC1). In turn, mTORC1 is inhibited by tuberous sclerosis complex 1/2 activation or p27 phosphorylation [184,185,186]. After ULK complex formation, Beclin-1 (Atg6) is recruited by the ER-resident soluble N-ethylmaleimide-sensitive factor attachment protein receptor (SNARE) protein syntaxin 17 (STX17) [187] and phosphorylated by ULK1 to initiate the formation of its complex, which is composed of Beclin-1, Atg14L, p150, and Vps34 (class III PI3K subunits) [188,189]. This complex produces phosphatidylinositol-3-phosphate [190], which recruits Atg18 (WD repeat domain phosphoinositide-interacting protein 1/2 in mammals) [191]. The Atg9–Atg2–Atg18 complex acts as a cycling system and contributes to phagophore expansion and elongation via membrane addition [192]. Then, phagophore spread continues via the ubiquitin-like Atg5–Atg12–Atg16 and Atg-7–Atg3–Atg8/LC3 complexes, which are required for the generation of membrane-associated LC3-II and the stabilization of the nascent autophagosome membrane [193]. Nascent LC3 is first processed by the protease Atg4 and cytosol-resident LC3-I, which is activated by Atg7 and transferred to Atg3. Finally, the Atg12–Atg5–Atg16 complex conjugates LC3-I with phosphatidylethanolamine to form LC3-II [194,195], which, together with p62 and other cargo receptors, is indispensable for the selectivity of autophagy [196]. Eventually, the expanding membrane closes around its cargo to form a complete autophagosome, which may require charged multivesicular body protein 2A (an endosomal sorting complex required for transport III subunit), vacuolar protein sorting-associated protein (VPS) 4 (an AAA ATPase), and VPS37A [197,198].

Autophagosomes migrate to the lysosomes and fuse with them to form autolysosomes for cargo degradation. The cytoskeleton and proteins such as STX17, Rab7, lysosomal-associated membrane protein (LAMP)1/2, [20], synaptosomal-associated protein 29 (SNAP29), and vesicle-associated membrane protein 8 (VAMP8) [199,200] seem to be involved in this migration. The deacetylation of STX17 by the inactivation of the cyclic adenosine monophosphate response element-binding protein promotes the formation of the STX17–SNAP29–VAMP8 SNARE complex, leading to autolysosome formation [199,200]. LAMP2 appears to be key to proper STX17 function [201]. Another key process for autophagy regulation is lysosomal biogenesis, which appears to be regulated by TFEB [202].

#### 3.3.2. Mitophagy

Mitophagy is a selective form of macroautophagy that mediates mitochondrial degradation in the autolysosomes. In type-1 mitophagy, small (0.2–0.3-μm) pre-autophagosomal structures grow into cup-shaped phagophores that envelop and sequester mitochondria into mitophagosomes, often in coordination with mitochondrial fission. In type 2 mitophagy, cup-shaped phagophores are not formed; rather, LC3 aggregates sequester individual mitochondria into mitophagosomes. In both types of mitophagy, mitophagosomes form, acidify, fuse with lysosomes, and degrade their contents [154,156]. While type 1 is primarily related to physiological mechanisms, such as nutrient deprivation, type 2 is related to (and activated by) sensors of mitochondrial damage, such as those caused by oxidative stress [203]. The main oxidative-stress-dependent pathway is that of phosphatase and the tensin homolog-induced putative kinase 1 (PINK1)/Parkin/p62 [203,204,205].

PINK1 is a serine/threonine kinase that translocates to the outer mitochondrial membrane, where it is stabilized by a low mitochondrial transmembrane potential and senses mitochondrial depolarization [206]. It recruits and phosphorylates Parkin (a ubiquitin E3 ligase) [207], which ubiquitylates several proteins on the outer mitochondrial membrane, including voltage-dependent anion channels. p62 recognizes ubiquitylated proteins [204] which trigger their degradation through the lysosome pathway via autophagy via LC3-II interaction [208,209].

#### 3.3.3. Acute Alcohol Intake and Autophagy in Hepatocytes

**Figure 2:**
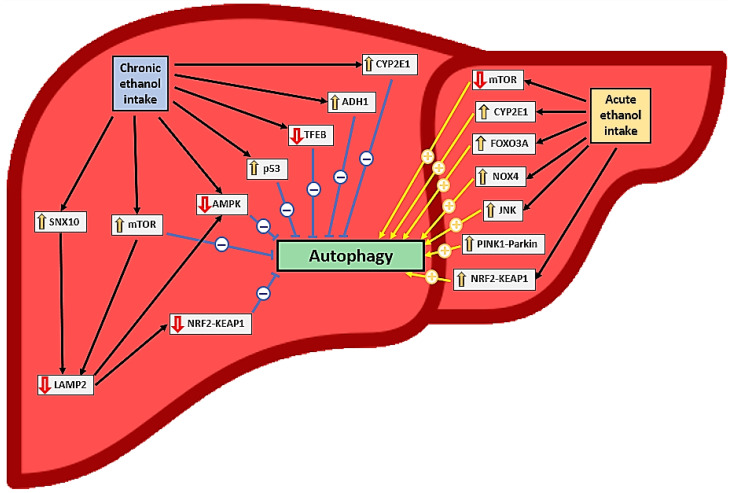
Effects of ethanol consumption on liver autophagy and relationships to oxidative stress. The symbol “↑” represents an increase in expression or activation while the symbol “↓“ represents a decrease in expression or inactivation.

Current evidence clearly indicates that acute alcohol intake and exposure activate autophagy in hepatocytes in vivo and in vitro, respectively [12,32,33,37,38,42,44,46,47,49] (Table 1 and Table 3, Figure 2); a reduction in autophagy after ethanol intake has been documented in only one study [48]. This activation depends on ethanol oxidation, ROS generation [37], mTOR [38], and proteasome inhibition [49] but not on acetaldehyde [32]. In this context, the induction of autophagy is probably more closely related to CYP2E1 pathway activation than to NOX4 [32], JNK [36], and forkhead box O3a (FOXO3A) [45,46] activation (Figure 2). Autophagy in hepatocytes appears to play a protective role against oxidative stress, steatosis, and inflammation caused by acute alcohol consumption [34,36,38,47]. Antioxidants may help reduce oxidative stress, but they may also indirectly reduce autophagy induction [32], mitigating its protective role.

The nuclear factor erythroid 2-related factor 2 (Nrf2)-mediated antioxidant response to acute alcohol exposure in hepatocytes is controversial; it may induce p62-dependent autophagy, which may have a protective role by increasing chaperone-mediated autophagy [35]. However, some researchers have found that this activation may worsen the negative effects of alcohol ingestion by activating ferroptosis [33] (Figure 2). Alcohol exposure also activated type 2 mitophagy in hepatocytes through the PINK1-Parkin-LC3 pathway and mitochondrial depolarization [12,42] (Table 1 and Table 3, Figure 2).

#### 3.3.4. Acute Alcohol Intake and Autophagy in Other Cell Types

No study included in our systematic review evaluated autophagy in Kupffer cells (KCs) after acute ethanol intake or exposure. A model in which HSCs were acutely exposed to ethanol revealed increased autophagy, oxidative stress, and cellular activation, with the former regulating the latter two processes via the Nrf2–Keap1–antioxidant response element pathway [62]. The inhibition of autophagy reversed HSC activation and suppressed oxidative stress [62] (Table 5).

#### 3.3.5. Chronic Ethanol Intake and Autophagy in Hepatocytes

However, the effect of chronic ethanol intake on autophagy in hepatocytes remains unclear. Some studies have shown that chronic ethanol intake or exposure activates autophagy in these cells [47,52,58] and has a protective role similar to acute intake [47]. In these studies, CYP2E1 expression or the reduction of proteasome activity appeared to induce autophagy (e.g., through an increase in LC3 level) [41,49]. Babuta et al. [52] observed decreased LAMP1/2 and lysosomal marker expression, despite increased autophagy [with reduced mTOR and Ras homolog enriched in brain (Rheb) and increased LC3-II expression] in mouse hepatocytes after chronic ethanol intake.

In contrast, chronic alcohol consumption reduced autophagy in hepatocytes in several studies [31,35,39,40,40,50,51,52,53,54,55,56,57,59] (Table 2 and Table 4, Figure 2). This effect was accompanied by decreased levels of LC3 [39,40,59], Beclin-1 [59], Atg5 [54], Atg7 [57], Atg3, Atg12 [59], TFEB [55], AMPK [59], and LAMP1/2 [50,51,52] and increased p62 levels [40,54,57,59], mTOR pathway activation [50,55], and iron overload [59]. This reduction in autophagy has negative effects (e.g., as hepatotoxicity, steatosis, and oxidative stress) and appears to be related mainly to CYP2E1 [40,57] and ADH1 [39] activation. Ethanol, acetaldehyde, and hepatic free fatty acids may reduce autophagy in hepatocytes after chronic ethanol treatment [39,50]. This treatment also appears to induce ER stress [50,51,59].

The inhibition of chronic intake exposure-induced autophagy in hepatocytes and the development of steatosis, inflammation, and oxidative stress can be attenuated by ALDH2 expression [39], CYP2E1 inhibition [40], and LAMP2A overexpression [50,51]. LAMP2A plays a protective role by activating the Nrf2 and AMPK pathways by increasing chaperone-mediated autophagy [35]. LAMP2 suppression, which is probably mTORC1-dependent, has been observed in patients with severe alcoholic hepatitis [50] (Table 2 and Table 4, Figure 2). Mitophagy in hepatocytes is suppressed after chronic ethanol exposure, probably due to the upregulation of the DNA-dependent protein kinase catalytic subunit and p53 activation [53]. In this context, AMPK has been shown to enhance mitophagy via the Nrf2–ubiquinol–cytochrome C reductase core protein 2 pathway [31].

Mice lacking proteins required for LC3, Atg5, and Atg7 lipidation show different vulnerabilities to acute and chronic alcohol exposure. For instance, Atg5-knockout (KO) mice are more susceptible to liver damage with acute alcohol treatment, whereas Atg7-KO mice are more sensitive to liver damage after chronic plus binge ethanol intake [43]. Atg5 inhibition appears to improve chronic ethanol consumption-induced liver damage [43]. Similarly, CYP2E1 activation is associated with increased autophagy after acute ethanol intake [43] but reduced autophagy after chronic ethanol intake (Figure 2). The mechanisms involved in these differences are not adequately understood [43].

Nevertheless, autophagy activation seems to protect against liver damage induced by chronic and acute ethanol exposure [43] as well as increased apoptosis [33,54,59] (Table 2 and Table 4, Figure 2). In addition, selective autophagic pathways, such as mitophagy, which allows hepatocytes to adapt to chronic ethanol exposure, may improve hepatocyte biology [31,44,53] (Table 2 and Table 4).

#### 3.3.6. Chronic Ethanol Intake and Autophagy in Other Cell Types

In KCs exposed chronically to alcohol, autophagy seems to have a protective role associated with decreased Myeloid differentiation factor 2/Toll-like receptor 4 expression [56] and the mediation of the anti-inflammatory and anti-steatogenic effects of the cannabinoid 2 receptor [63]. The inhibition of autophagy in macrophages after chronic ethanol treatment appeared to increase ethanol-induced liver damage, inflammation, ROS generation, interferon regulatory factor 1 accumulation, and the induction of hepatic C-C motif chemokine ligand 5 and C-X-C motif chemokine ligand 10 expression [60,61] (Table 5).

HSCs from ethanol-fed mice had an increased UPR, which triggered autophagy and induced an Nrf2-mediated antioxidant response under ER stress conditions [146]. The IRE1α pathway blockade significantly reduced autophagy activation in a p38 MAPK-dependent manner, thereby reducing the fibrogenic response [64] (Table 5).

### 3.4. Alcohol-Induced Organ–Organ Crosstalk and Autophagy

Information on the autophagic pathways involved in organ–organ crosstalk in ALD is scarcely available, and the studies that have been performed have focused mainly on the crosstalk between adipose and liver tissue. Rodriguez et al. [210] showed that the ablation of the mTOR (Raptor) pathway in adipocytes (but not hepatocytes) contributed to acute alcohol treatment-induced liver damage with increased inflammation, suggesting the implication of adipose tissue in the development of alcoholic steatohepatitis. Consistently, Li et al. [153] reported exacerbated alcohol-induced hepatic steatosis in adipocyte-specific Raptor–KO mice. Adipocyte-specific Atg5–KO mice had increased circulating levels of fibroblast growth factor 21 (FGF21) and adiponectin and were resistant to chronic alcohol treatment-induced adipose tissue atrophy and liver damage [211]. Although this area of research, particularly in the context of ALD, is relatively new, current evidence suggests that autophagy modulates the crosstalk between adipose tissue and the liver by controlling the synthesis of FGF21 and adiponectin. Such autophagy-mediated crosstalk has been detected in other liver diseases, such as non-alcoholic fatty liver disease, in which autophagy inhibition in white adipose tissue after four months of a high-fat diet ameliorated liver pathology in mice [212].

### 3.5. Autophagy-Targeting Treatments for ALD

Effective therapies for ALD are lacking, and given its important role in this disease, autophagy is a potential therapeutic target. The roles of several pharmacological agents in the prevention or amelioration of ALD through autophagic modulation have been examined (Table 6). Most of these studies have been performed using mouse models, although other animal models (i.e., rat and zebrafish) have also been used. The mTOR pathway is one of the most relevant potential therapeutic targets in this context. The activation of mTOR and AMPK signaling is involved in ethanol-induced autophagy under oxidative stress [14,213]. Rapamycin (also known as sirolimus) is a lipophilic macrolide antibiotic that was first isolated from *Streptomyces hygroscopicus* and has been shown to inhibit mTORC1, thereby reversing alcohol-induced mTOR activation and attenuating related liver damage [15,40,51]. Torin 1 is a selective ATP-competitive small molecule that inhibits the mTOR pathway through direct inhibition of the mTORC1 and mTORC2 complexes [55,105], and it may also ameliorate ALD. Upstream of mTOR, the pathway can be modulated by the inhibition of AMPK. The activation of AMPK inhibits mTOR-dependent signaling through different molecules, such as calcitriol (the active form of vitamin D) [69] and palmatine (a protoberberine alkaloid found in several plants) [87]. Although no clinical trials have been performed to study the effects of these drugs on ALD, studies are being undertaken to examine their effects on Sjögren syndrome (ClinicalTrials.gov identifier: NCT05605665) and Alzheimer’s disease (ClinicalTrials.gov identifier: NCT04629495). The possibility of rapamycin delivery via nanoparticles, which may reduce side effects and have been shown to ameliorate metabolic fatty liver disease in a mouse model [214], adds to the interest in testing the effect of this drug on ALD.

Ethanol intake activates FOXO3a, which transcriptionally regulates several autophagy genes [46]. The SIRT1/FOXO pathway is involved in the alleviation of chronic alcoholic liver damage by preventing fat accumulation and reducing ROS production, inflammation, and cell death [215]. Modulators of autophagy through this pathway, such as resveratrol, quercetin, and salvianolic acid A [46,90,91,92,93,94,96,97,98], have shown promise for the treatment of ALD in animal models. We are not aware of a clinical trial examining the effects of these products, but potential interest due to the promising experimental results and favorable side effect profiles, particularly with resveratrol, is attenuated by the lack of efficacy against non-alcoholic fatty liver disease [216].

Alcohol-induced autophagy can also be suppressed by antioxidants such as N-acetylcysteine (NAC) [38,40]. NAC and the CYP2E1 inhibitor chlormethiazole [40,72,73] appear to attenuate the toxic effects of ethanol in the liver [40]. NAC has been tested in a clinical trial conducted in patients with alcoholic hepatitis; although infections were less frequent and 1-month mortality was reduced in the prednisolone-NAC group relative to the prednisolone-only group, other side effects and the 6-month survival rate were similar across the two groups [217]. Another clinical trial examining the efficacy of NAC against alcoholic hepatitis is ongoing (ClinicalTrials.gov identifier: NCT03069300), given that the body of evidence provides a rationale for the use of this drug in patients with ALD and/or alcohol use disorders [218].

Other products, such as zinc and carbamazepine, appear to be involved in autophagy activation after ethanol intake or exposure; thus, they could be tested as potential treatments for ALD. Interestingly, zinc exposure stimulates autophagy, with an additive effect of co-stimulation with ethanol for 24 h [107]. Carbamazepine, a mood-stabilizing drug, induces autophagy by reducing the level of intracellular inositol [47]. The administration of Nrf2 activators, such as sulforaphane [102] from vegetables of the genus *Brassica* and glycycoumarin from *Glycyrrhiza uralensis* [81], is also of potential interest in the treatment of this disease. Data from animal models suggest that these agents improve alcohol-induced liver steatosis and oxidative stress and promote autophagy (Table 6). The activation of the Nrf2 pathway appears to protect against alcohol-induced liver fibrosis and hepatotoxicity, whereas Nrf2 knockdown is associated with increased alcohol-induced hepatocyte damage [81,219,220].

## 4. Discussion

Acute ethanol consumption increases autophagy activation in the liver, whereas chronic alcohol intake or exposure decreases autophagy activation. The mechanisms underlying this difference have not yet been fully elucidated. Increased autophagy in hepatocytes and KCs seems to have a protective role against the pathogenesis of alcohol-related liver damage and ALD, and the inhibition of autophagy makes hepatocytes susceptible to hepatotoxicity, steatosis, and oxidative stress. Autophagy leads to HSC activation and oxidative stress in these cells.

Most studies in this field have examined the classical components of autophagic pathways (e.g., p62, LC3, and Beclin 1) in liver cells; expanding the scope of research to examine other components is desirable. For instance, autolysosome formation, a key step in autophagy, has not been analyzed in detail in the context of ALD. In addition, autophagy in different organs and tissues, especially in cells of the immune system, needs to be assessed because of the involvement of these cells and tissues in inflammation and oxidative stress, which play key roles in ALD development. Selective autophagy pathways such as mitophagy and lipophagy should also be analyzed further. Autophagy-related organ–organ crosstalk in ALD has received little research attention despite the growing interest in and potential of this field. Regarding clinical applications, autophagy-targeting therapies have not been tested successfully in clinical trials, although data from animal and in vitro models suggest potential roles for several drugs, including those involved in the mTOR pathway and antioxidants that modulate autophagy. Although a large number of drugs could potentially be useful in ALD treatment, based on basic research, the potential difficulties of carrying out large clinical trials, particularly those not funded by drug companies, could prove difficult to navigate, in spite of the fact that autophagy targeting may have protective effects against several liver diseases [47]. We hope to see expansion in this area in the near future.

It is important to acknowledge that the variations observed in the definition of acute or chronic ethanol intake or exposure, as well as the diverse experimental protocols employed across the included studies, have posed challenges in clearly elucidating the underlying mechanisms associated with acute and chronic ethanol exposure and have prevented us from employing a meta-analysis or other numerical approaches to demonstrate the extent of variability (e.g., classic coefficient of variation or robust coefficient of variation estimators [221].

## 5. Conclusions

In conclusion, autophagy activation in hepatocytes and KCs appears to have a protective role against alcohol-induced liver damage and oxidative stress. However, further research is needed to elucidate these mechanisms and develop potential clinical applications.

## Figures and Tables

**Table 1:** Studies included in our systematic review that focused on the effects of acute ethanol exposure on cell models.

First Author, Year	Models and Methods	Summary of Effects
Lu et al., 2021 [31]	AML-12 cells treated with 200 mM ethanol over 24 h.	AMPK enhances mitophagy in hepatocytes, and the AMPK–NFE2L2–UQCRC2 axis regulates liver mitophagy.
Chen et al., 2021 [32]	Huh7 cells and murine primary hepatocytes treated with 50–100 mM ethanol over 24 h.	Acute ethanol exposure induces NOX4 and CYP2E1 overexpression and significantly increases autophagy. Antioxidants efficiently block CYP2E1- and NOX4-mediated autophagy induction.
Zhao et al., 2021 [33]	HepG2 cells treated with 0, 100, 200, 300, 400, and 500 mM alcohol over 12 h.	Alcohol induces reversible ferroptosis, which is significantly reduced by ferrostatin-1. Inhibiting autophagy protects HepG2 cells against alcohol-induced ferroptosis by activating the p62−Keap1−Nrf2 pathway.
Liu et al., 2019 [34]	HepG2 cells treated with 200 mmol/L ethanol for 12 h.	Autophagy is inhibited in ethanol-treated HepG2 cells. ALR-expressing HepG2 cells have increased survival rates, improved mitochondrial membrane potential, and increased ATP levels after ethanol treatment. This protection is associated with the upregulation of autophagy markers and downregulation of p62 and mTOR phosphorylation.
You et al., 2018 [35]	Mouse hepatocytes treated with 80 mM ethanol for 6 h.	SNX10 deficiency upregulates LAMP2A expression and CMA activation via Nrf2 and AMPK signaling in vitro, significantly ameliorating ethanol-induced liver damage and hepatic steatosis.
Mahli et al., 2015 [36]	Primary human hepatocytes and HepG2 cells treated with 50 mM alcohol over 16–24 h.	Alcohol and steatosis increase CYP2E1 levels and activity, lipid peroxidation, oxidative stress, pro-inflammatory gene expression, and autophagy via the CYP2E1 and JNK pathways. Autophagy improves the effects of alcohol on lipid accumulation and inflammatory gene expression in liver cells.
Thomes et al., 2013 [37]	Hep G2 cells treated with 50 mM ethanol over 24 h.	Ethanol treatment increases LC3-II expression and decreases its degradation in a dose-dependent manner depending on ADH and CYP2E1 expression. Blocking ethanol oxidation and ROS production prevents the enhancement of LC3-II expression. Direct exposure to acetaldehyde enhances LC3-II content.
Ding et al., 2010 [38]	Murine hepatocytes and HepG2 cells treated with 40, 80, and 160 mM ethanol for 24 h.	Ethanol-induced autophagy requires ethanol metabolism, ROS generation, and mTOR signaling inhibition in vitro. It is selective for cells with damaged mitochondria and accumulated lipid droplets (but not long-lived proteins) and protects cells from ethanol’s toxic effects. Increasing autophagy reduces acute ethanol hepatotoxicity and steatosis.

AMPK, adenosine monophosphate-activated protein kinase; NFE2L2, nuclear factor erythroid 2-related factor 2; UQCRC2, ubiquinol–cytochrome C reductase core protein 2; Huh7, human hepatoma-derived 7 cell line; NOX4, nicotinamide adenine dinucleotide phosphate oxidase 4; HepG2, human epidermoid carcinoma strain 2; CYP2E1, cytochrome P450 2E1; Keap1, Kelch-like associ-ated a protein 1; Nrf2, nuclear factor erythroid 2-related factor 2; ALR, liver regeneration-associated protein; LAMP2A, lysosomal-associated membrane protein 2A; CMA, chaperone-mediated autophagy; JNK, c-Jun N-terminal kinase; LC3-II, microtubule-associated protein light chain 3 II; mTOR, mammalian target of rapamycin complex 1.

**Table 2:** Studies included in our systematic review that focused on the effects of chronic ethanol exposure on cell models.

First Author, Year	Models and Methods	Summary of Effects
Guo et al., 2015 [39]	HepG2 cells treated with 100 mM ethanol for 4 days.	Ethanol and acetaldehyde increase IL-6 and IFN-γ levels and suppress autophagy in ADH1-expressing HepG2 cells. Lysosomal inhibitors mimic ethanol-induced p62 accumulation.
Wu et al., 2012 [40]	HepG2 cells treated with 100 mM ethanol for 8 days.	Inhibiting autophagy enhances ethanol hepatotoxicity, steatosis, and oxidative stress in CYP2E1-expressing HepG2 cells. These cells show increased fat accumulation and oxidative stress but decreased autophagy. The antioxidant N-acetylcysteine and CYP2E1 inhibition blunt these effects.
Wu et al., 2010 [41]	HepG2 cells treated with 50, 100, and 150 mM ethanol for 4–5 days.	CYP2E1-expressing HepG2 cells have exacerbated lipid and TG accumulation and increased p62 levels. HepG2 cells show increased autophagy. Inhibiting autophagy increases lipid accumulation and TG levels in HepG2 cells and, to a lesser extent, in CYP2E1-expressing HepG2 cells. Ethanol induces CYP2E1 activity and oxidative stress in CYP2E1-expressing HepG2 cells.

HepG2, human epidermoid carcinoma strain 2; IL-6, interleukin 6; IFN-γ, interferon-γ; ADH1, alcohol dehydrogenase 1; CYP2E1, cytochrome P450 2E1; TG, triglycerides.

**Table 3:** Studies included in our systematic review focusing on the effects of acute ethanol intake and exposure on animal models.

First Author, Year	Models and Methods	Summary of Effects
Samuvel et al., 2022 [42]	Male C57BL/6 mice gavaged one dose ethanol (2–6 g/kg in normal saline, 20 µL/g body weight).	Acute ethanol treatment induces dose-dependent mitochondrial depolarization, leading to type 2 mitophagy sequestration (probably through the PINK1–Parkin pathway) and subsequent lysosomal processing.
Chen et al., 2021 [32]	Male C57BL/6 mice gavaged a total of 4.5 g/kg (body weight) of ethanol over 24 h.	Acute ethanol exposure induces autophagy and ROS-generating CYP2E1 and NOX4 enzymes. NOX4 and CYP2E1 overexpression significantly increases autophagy. Ethanol and H_2_O_2_ (but not acetaldehyde) induce autophagy in primary mouse hepatocytes. Antioxidants efficiently block CYP2E1- and NOX4-mediated autophagy induction.
Yan et al., 2019 [43]	Male/female C57BL/6 mice; ethanol (5 g/kg body weight) gavage for 16 h.	Atg5-KO mice are more susceptible to acute alcohol treatment, but liver damage is unexpectedly improved with the chronic plus binge model.
Liu et al., 2019 [34]	Male C57BL/6 mice gavaged a total of 10 mL/kg 55% ethanol over 12 h.	Mice overexpressing ALR have less liver damage with alcohol exposure, associated with the upregulation of autophagy markers and downregulation of p62 and mTOR phosphorylation. Autophagy is inhibited in ALR-KO mice.
Eid et al., 2016 [12]	Adult male Wistar rats given one intraperitoneal ethanol dose (40% *v*/*v*, 5 g/kg) over 24 h.	Ethanol induces a low level of hepatocyte apoptosis but enhances mitophagic vacuole formation (increased LC3 puncta formation and co-localization of Parkin and LC3). PINK1 and Parkin are located around damaged mitochondria in the hepatocytes of ethanol-treated rats with an enhanced formation of mitochondrial spheroids.
Williams et al., 2015 [44]	Male C57BL/6J mice gavaged with a total of 4.5 g/kg of ethanol per kg of body weight over 16 h.	Parkin prevents liver damage in an acute alcoholic model. Ethanol-fed Parkin-KO mice exhibit severe mitochondrial damage; reduced mitophagy, β-oxidation, mitochondrial respiration; and cytochrome C oxidase activity.
Manley et al., 2014 [45]	Male C57BL mice gavaged with a total of 4.5 g/kg ethanol per kg of body weight ethanol over 16 h.	FXR-KO mice have exacerbated hepatotoxicity, steatosis, decreased essential autophagy-related gene expression, increased Akt activation, and decreased FOXO3a activity. Ethanol treatment induces hepatic mitochondrial spheroid formation in FXR-KO, but not WT, mice.
Ni et al., 2013 [46]	Male C57BL/6 mice gavaged with a total of 4.5 g/kg ethanol per kg of body weight ethanol over 6, 12, and 16 h.	Ethanol-fed mice have increased mRNA and protein levels of autophagy-related genes in hepatocytes and FOXO3a activity. Suppressing FOXO3a activity in hepatocytes inhibits autophagy-related gene expression and enhances cell death, steatosis, and liver damage. A SIRT1 agonist enhances ethanol-induced autophagy-related gene expression by increasing FOXO3a deacetylation.
Lin et al., 2013 [47]	Intraperitoneal ethanol (33%, *v*/*v*, 1.2 g/kg body weight) injection over 10 min.	Macroautophagy is activated under acute conditions. Hepatic steatosis and liver damage are exacerbated by autophagy inhibition and alleviated by autophagy activation.
Yang et al., 2012 [48]	Male SV/129 and C57BL/6J mice intraperitoneally injected with ethanol (0.93 g/kg body weight) and later gavaged three times (total: 3.75 g/kg body weight) over 18 h.	WT mice show CYP2E1 activation; increased oxidative stress, JNK signaling, and SREBP expression; and decreased autophagy. Acute alcohol-induced fatty liver and oxidative stress are blunted in CYP2E1-KO and JNK inhibitor-treated mice. N-acetylcysteine decreases acute alcohol-induced oxidative stress, JNK activation, and steatosis but not CYP2E1 activation. Acute alcohol-induced fatty liver is the same in JNK1 and JNK2-KO mice as in WT mice.
Thomes et al., 2012 [49]	Female C57Bl/6 mice gavaged a total of 6 g/kg body weight ethanol over 12 h; precision-cut liver slices treated with 50 mM ethanol over 12–24 h.	Acute ethanol administration elevates autophagosomes without affecting hepatic proteasome activity. Liver slices show inhibited proteasome activity and enhanced autophagosome expression depending on ethanol oxidation.
Ding et al., 2010 [38]	Male C57BL/6 mice gavaged with a total of 4.5 g/kg ethanol per kg of body weight over 16 h.	Ethanol-induced autophagy in vivo requires ethanol metabolism, ROS generation, and mTOR signaling inhibition. Increasing autophagy reduces acute ethanol hepatotoxicity and steatosis.

PINK1, tensin homolog-induced putative kinase 1; NOX4, nicotinamide adenine dinucleotide phosphate oxidase 4; CYP2E1, cytochrome P450 2E1; Atg 5, antithymocyte globulin 5; LC3, microtubule-associated protein light chain 3; ALR, liver regeneration-associated protein; mTOR, mammalian target of rapamycin complex 1; KO, knockout; WT, wild-type; ALR, augmenter of liver regeneration; FXR, Farnesoid X receptor; akt, protein kinase B; FOXO3a, forkhead box O3a; SIRT1, sirtuin-1; JNK1/2, c-Jun N-terminal kinase 1/2; SREBP, sterol regulatory element-binding protein.

**Table 4:** Studies included in our systematic review that focused on the effects of chronic ethanol intake and exposure on animal models.

First Author, Year	Models and Methods	Summary of Effects
Lu et al., 2021 [31]	NIAAA model with male C57BL/6J mice.	Alcohol induces low UQCRC2 expression, which is alleviated by AMPK. The AMPK–NFE2L2–UQCRC2 axis regulates liver mitophagy.
Guo et al., 2021 [50]	Lieber–DeCarli model with male C57BL/6J mice. Liver tissues from patients with alcoholic hepatitis were examined.	Palmitic acid in alcohol-fed mice induces ER stress and mTORC1-dependent LAMP2 suppression. mTORC1 signaling induction and CHOP were detected in patient livers.
Guo et al., 2021 [51]	Lieber–DeCarli model with male C57BL/6J mice. Liver tissues from patients with alcoholic hepatitis were examined.	The pathogenesis of ALD is mediated by hepatic free fatty acid accumulation, which suppresses the LAMP2–autophagy flux pathway through ER stress signaling.
Babuta et al., 2019 [52]	Lieber–DeCarli and NIAAA models with female C57BL/6 mice. Liver tissues from patients with alcoholic hepatitis were examined.	Chronic alcohol intake impairs autophagy in livers, decreasing mTOR and Rheb and increasing Beclin-l and Atg7 expression, disrupting autophagy at the lysosomal level by decreasing LAMP1 and LAMP2 expression. Alcohol increases miR-155 targeting of mTOR, Rheb, LAMP1, and LAMP2. miR-155-deficient mice are protected from alcohol-induced autophagy disruption and have attenuated exosome production. LAMP1/2 downregulation increases exosome release in hepatocytes in the presence and absence of alcohol.
Zhou et al., 2019 [53]	Female C57BL mice fed liquid diet with 4% (vol/vol) alcohol for 16 weeks.	Chronic alcohol consumption increases DNA-PKcs in the liver, leading to liver damage and mitochondrial dysfunction through p53 activation and defective mitophagy.
Yan et al., 2019 [43]	NIAAA model with male/female C57BL/6 mice.	Mice lacking the Atg7 gene had more liver damage from alcohol and were more susceptible to chronic plus binge drinking. Long-term autophagy deficiency worsened the liver’s response to alcohol.
Menk et al., 2018 [54]	Lieber–DeCarli model with male Wistar rats for 12 weeks.	Chronic alcohol consumption causes stress in the liver, impairs autophagy-related gene expression, disrupts autophagic flux, and increases apoptosis in the liver.
You et al., 2018 [35]	Lieber–DeCarli model with male FVB and C57BL/6J mice for 4 weeks.	SNX10 deficiency increases LAMP2A expression and CMA activation, improving liver damage and fat accumulation caused by alcohol through the activation of Nrf2 and AMPK signaling.
Chao et al., 2018 [55]	NIAAA model with male C57BL/6N mice. Liver tissues from patients with alcoholic hepatitis were examined.	Alcohol-fed mice had lower levels of TFEB, decreased lysosome and autophagy activity, and increased mTOR activation. Activating the TFEB pathway reversed these effects. Mice lacking TFEB or both TFEB and TFE3 had more severe liver damage from alcohol. Patient liver tissues had lower levels of nuclear TFEB than control tissues.
Kong et al., 2017 [56]	Lieber–DeCarli model with male C57BL6 mice for 16 days with intraperitoneal LPS injection (10 mg/kg) on the final day.	Alcohol-fed mice experienced fat accumulation, liver damage, and increased inflammation. LPS worsened alcohol-induced oxidative stress and reduced autophagy activity.
Williams et al., 2015 [44]	NIAAA model with male C57BL/6J mice.	Parkin prevented liver damage in chronic alcohol-fed mice. Mice lacking Parkin had severe mitochondrial damage, reduced mitophagy and mitochondrial function, and an impaired ability to adapt to alcohol treatment.
Guo et al., 2015 [39]	Female FVB mice fed a 4% (vol/vol) alcohol liquid diet for 6 weeks.	Chronic alcohol intake causes liver damage, disturbed fat metabolism, increased inflammation and oxidative stress, and decreased autophagy. Expressing the ALDH2 gene reduced these effects. Lysosomal inhibitors had the same effects as alcohol on p62 accumulation.
Lu and Cederbaum, 2015 [57]	Lieber–DeCarli model with male SV/129 mice for 4 weeks.	Inhibiting autophagy increased liver damage and fat accumulation in mice with normal or increased CYP2E1 levels but not in mice lacking CYP2E1. Autophagy did not affect CYP2E1 activity or induction by alcohol. Mice with normal or increased CYP2E1 levels had decreased autophagy-related gene expression and increased p62 levels.
King et al., 2014 [58]	Lieber–DeCarli model with male C57BL/6J mice.	Alcohol-treated mice experienced fat accumulation, increased autophagy, decreased mitochondrial function, and increased CypD levels. Their mitochondria were more sensitive to damage than those of mice lacking CypD. CypD deficiency impaired autophagy but did not prevent fat accumulation caused by alcohol.
Tan et al., 2013 [59]	Male C57/BL6 mice fed 5–20% (vol/vol) ethanol and a high-fat diet for 8 weeks.	Mice lacking the HFE gene had liver damage, fibrosis, and increased cell death. Iron overload in these mice caused stress responses and impaired autophagy-related gene expression and activity.
Lin et al., 2013 [47]	Lieber–DeCarli model with C57BL/6 mice for 4 weeks.	Macroautophagy was activated during chronic alcohol consumption. Inhibiting autophagy worsened liver damage and fat accumulation, while activating autophagy improved these conditions.
Wu et al., 2012 [40]	Male SV129 mice gavaged with a total of 3 g/kg body weight ethanol over 4 days.	Alcohol treatment caused liver damage, increased CYP2E1 levels, and oxidative stress in mice with normal or increased CYP2E1 levels but not in mice lacking CYP2E1. Alcohol impaired autophagy in mice with increased CYP2E1 levels. Inhibiting autophagy worsened alcohol-induced liver damage, fat accumulation, and oxidative stress in these mice.
Thomes et al., 2012 [49]	Lieber–DeCarli model with GFP-LC3 tg mice for 4–6 weeks.	Chronic alcohol-fed mice had reduced proteasome activity and increased autophagy markers in liver cells. Inhibiting the proteasome further increased autophagy markers.

UQCRC2, ubiquinol–cytochrome C reductase core protein 2; AMPK, adenosine monophosphate-activated protein kinase; NFE2L2, nuclear factor erythroid 2-related factor 2; mTORC1, mammalian target of rapamycin complex 1; LAMP1/2/2A, lysosomal-associated membrane protein 1/2/2A; CHOP, C/EBP Homologous Protein; Rheb, Ras homolog enriched in brain; Atg7, antithymocyte globulin 7; miR, micro-RNA; DNA-PKCs, DNA-dependent protein kinase catalytic subunit; SNX10, sorting nexin-10; CMA, chaperone-mediated autophagy; NRF2, nuclear factor erythroid 2-related factor 2; TFEB, transcription factor EB; TFE3, transcription factor binding to IGHM enhancer 3; LPS, lipopolysaccharide; ALDH2, aldehyde dehydrogenase 2; CYP2E1, cytochrome P450 2E1; CypD, cyclophilin D; HFE, hemochromatosis; GFP, green fluorescent protein; LC3, microtubule-associated protein light chain 3.

**Table 5:** Studies included in our systematic review that focused on effects of ethanol intake or exposure in non-hepatocyte liver cells.

First Author, Year	Models and Methods	Summary of Effect
Liang et al., 2019 [60]	Lieber–DeCarli model with female C57BL/6 mice and intraperitoneal LPS injection (1 mg/kg) on the final day.	Chronic alcohol feeding increased liver damage and inflammation in mice lacking the Atg7 gene in immune cells and increased inflammatory gene expression in normal mice. Mice lacking Atg7 experienced impaired mitochondrial function, increased oxidative stress, and increased inflammation. Silencing p62 or deleting Atg7 caused the accumulation of IRF1 and increased inflammatory gene expression.
Ilyas et al., 2019 [61]	Female C57BL/6J mice fed 5% ethanol liquid diet for 21 days with intraperitoneal LPS injection (7.5 mg/kg) on the final day.	Mice lacking the Atg5 gene in immune cells had similar fat accumulation compared to normal mice when fed alcohol but had increased liver damage, inflammation, and cell death. Blocking the IL-1 receptor reduced alcohol-induced inflammation.
Xie et al., 2018 [62]	HSC-T6 cells treated with 100 mmol/L alcohol.	The treatment increased autophagy and oxidative stress and activated HSCs. Inhibiting autophagy reversed HSC activation and reduced oxidative stress in HSCs. The Nrf2-Keap1-ARE pathway was involved in regulating HSC activation and oxidative stress through autophagy.
Kong et al., 2017 [56].	RAW 264.7 cells treated with various alcohol doses for 48h plus LPS (100 ng/mL) for 6h.	The protective effects of autophagy are associated with decreased cellular MD2/TLR4 expression in RAW 264.7 cells.
Denaës et al., 2016 [63]	NIAAA model with C57BL/6N mice.	Mice lacking the CB2 gene in immune cells experienced worsened alcohol-induced inflammation and fat accumulation. Activating the CB2 receptor reduced alcohol-induced liver inflammation and fat accumulation in normal mice but not in mice lacking the ATG5 gene in immune cells. Macrophage autophagy mediated the protective effects of the CB2 receptor.
Hernández-Gea et al., 2013 [64]	Lieber–DeCarli model with C57/BL6 mice.	An increase in the UPR, as indicated by XBP1 mRNA splicing, triggered autophagy. The Nrf2-mediated antioxidant response was activated during ER stress. Blocking the IRE1α pathway in HSCs reduced their activation and autophagy activity, reducing fibrosis through a p38 MAPK-dependent mechanism.

Atg5/7, antithymocyte globulin 5/7; IRF1, interferon regulatory factor 1; IL-1, interleukin 1; HSC, hepatic stellate cell; Nrf2, nuclear factor erythroid 2-related factor 2; Keap1, Kelch-like associ-ated a protein 1; ARE, antioxidant response element; MD2, Myeloid Differentiation factor 2; TLR4, Toll-like receptor 4; CB2, cannabinoid 2; XBP1, X-box binding protein 1; IRE1α, inositol-requiring transmembrane ki-nase/endoribonuclease 1α; MAPK, mitogen-activated protein kinase.

**Table 6:** Potential therapeutic approaches for alcoholic liver disease by targeting autophagy pathways.

Drug	Pharmacological Classes	Experimental Model	Main Pathways Involved	Disease Prevention or Potential Benefits
AT extract [65]	Flavonoids, phenolic compounds, steroidal glycosides, coumarins.	Intragastric administration of ethanol (5 g/kg b.d., 7 days) or carbon tetrachloride ± AT extract (50 and 150 mg/kg/d) to mice, HepG2 and SK-Hep-1 cells exposed to ethanol.	Induction of autophagy through the activation of Nrf2 and MAPK and increased HO-1 levels.	Reduced liver damage and histopathological changes via increased antioxidant activity.
ACE [66]	Basidiomycete triterpenoids, flavonoids, fatty acids, amino acids.	Administration of white wine (9.52 g/kg, 56°, 2 weeks) and ACE (75, 225, and 675 mg/kg, 2 weeks) to mice.	Reduced Akt/ NF-κB signalling.	Reduced alcohol-induced hepatotoxicity, oxidative stress, and regulation of AST, ALT, oxidation-related enzyme, inflammatory cytokine, and caspase levels.
BBD [67]	Traditional Chinese medicine.	Mice gavaged with ethanol (50%, 5 g/kg), pretreated with BBD (0.125, 0.25, and 0.5 g/kg).	Induction of autophagy through increased NRF2 expression and suppression of CYP450 2E1 induction.	BBD reduced alcohol-induced steatosis, hepatic lipid peroxidation, antioxidant depletion, and oxidative stress.
BSE [68]	High levels of flavonoids and polyphenols.	Lieber–DeCarli model for 10 days with intraperitoneal injection of 31.5% ethanol on the last day and BSE (100 and 200 mg/kg/d) gavage, cultured hepatocytes.	Autophagy induction via AMPK activation.	BSE decreased hepatic lipid accumulation and inflammatory macrophage infiltration; in vitro, it induced hepatic β-oxidation and reduced fatty acid synthesis.
Calcitriol [69]	Active form of vitamin D.	In vitro, human L02 hepatocytes were pretreated with 100 nM calcitriol, then stimulated acutely with 300 nM ethanol.	Induction of autophagy through the AMPK/mTOR signaling pathway and upregulation of ATG16L1.	Calcitriol alleviated ethanol-induced cytotoxicity and apoptosis caused by oxidative stress and mitochondrial damage in hepatocytes.
CBD [70]	Antagonist of CB1/CB2 receptor agonists (negative allosteric modulator of CB1, inverse agonist of CB2).	Mouse acute binge drinking model with intraperitoneal CBD injection (5 mg/kg, q 12 h), HepG2 (E47) cells exposed to ethanol ± CBD.	Induction of autophagy through the blunted activation of the JNK/MAPK pathway.	CBD prevented ethanol-induced autophagy reduction and reduced oxidative stress and acute alcohol-induced liver steatosis in mice.
CBZ [47]	Antiepileptic.	Lieber–DeCarli model ± intraperitoneal CBZ (25 mg/kg), chloroquine (60 mg/kg), or rapamycin (2 mg/kg) injection.	Enhanced mTOR-independent autophagy.	CBZ alleviated hepatic steatosis and liver damage and improved insulin sensitivity.
Carvacrol [71]	Monoterpenoid phenol.	Mouse model of acute ethanol intake with carvacrol pretreatment (10 mL/kg).	Induction of autophagy, likely through the inactivation of p38, and inhibition of cytochrome p450.	Carvacrol reduced the TG content and ethanol-induced liver histopathological changes.
CMZ [40,72,73]	Thiazole derivative.	Chronic ethanol intake mouse model with CMZ (50 mg/kg), acute ethanol intake mouse model ± CMZ (50 mg/kg).	Induction of autophagy through the activation of the AMPK, MAPK, and PI3K/Akt/GSK3β pathways, and inhibition of CYP2E1.	CMZ suppressed chronic ethanol-induced oxidative stress and pro-inflammatory cytokine production, attenuated acute ethanol-induced fatty liver.
Cilostazol [74]	Selective phosphodiesterase III inhibitor.	Acute alcohol intake rat model ± intraperitoneal cilostazol (10 mg/kg/d for 4 days; primary rat hepatocytes were examined.	Autophagy induction via AMPK pathway activation.	Cilostazol protected hepatocytes from apoptosis in vivo and in vitro.
Corosolic acid [75]	Pentacyclic triterpene acid extracted from *Lagerstroemia speciosa*.	Chronic ethanol intake mouse model (intragastric, 60%; 4.5, 6.5, and 9 g/kg/d for 4 weeks) ± corosolic acid (20%, 4 mL b.d., 5–12 weeks). HepG2 cells and BRL-3A liver cells were examined.	Induction of autophagy through the activation of the AMPK pathway and reduction of ROS levels.	Corosolic acid ameliorated alcoholic liver damage, reduced histopathological changes in vivo, and decreased ethanol-induced ROS elevation.
DMY [76]	Bioactive flavonoid from *Ampelopsis grossedentata*.	Lieber–DeCarli mouse model (1% 2 d, 2% 2 d, 4% 7 d, and 4% 6 weeks) ± oral DMY (75 and 150 mg/kg/d).	Induction of autophagy through the activation of the Keap-1/Nrf2 pathway and upregulation of p62.	DMY attenuated ethanol induced hepatic enzyme release, lipid peroxidation, TG accumulation, proinflammatory cytokine elevation, and histopathological changes while alleviating IL-1β and IL-6 elevation and pathological changes.
TAX [77]	Dihydroflavone.	Acute ethanol intake mouse model (intragastric) ± TAX (1, 5, and 25 mg/kg), HepG2 cells exposed to ethanol and TAX.	Induction of autophagy via AMPK activation and upregulated SIRT1 expression.	TAX reduced liver damage and inhibited alcohol-induced lipid accumulation in mouse livers.
Fisetin [78]	Plant polyphenol flavonoid.	Lieber–DeCarli mouse model ± fisetin; human primary HSCs co-cultured with ethanol.	Activation of autophagy through the activation of SIRT1 and inhibition of Sphk1-mediated ER stress.	Fisetin inhibited ER stress and improved alcohol-induced liver damage and fibrosis through the suppression of HSC activation.
Fucoidan [79,80]	Long-chain sulfated polysaccharide from various brown algae species.	Mice gavaged with ethanol (56%: 6 [7] mL/kg for 4 weeks then 8 [9] mL/kg for 12 [16] weeks) with daily intragastric fucoidan (100 and 200 [150 and 300] mg/kg).	Induction of autophagy via AMPKα1, SIRT1, and p62/Nrf2/Keap1/SLC7A11 pathway upregulation.	Fucoidan inhibited alcohol-induced steatosis, inflammation, oxidative stress, and histopathological changes; reduced the serum ferritin level; and alleviated liver iron deposition.
GMC [81]	Coumarin extracted from licorice.	Chronic and acute ethanol gavage mouse models ± GMC.	Induction of autophagy through the activation of Nrf2 and p38.	GCM prevented acute and chronic ethanol-induced hepatic steatosis in vivo and alleviated oxidative stress.
Green tea extract [82]	Tea polyphenols.	Chronic ethanol intake mouse model (50%, 15 mL/kg, intragastric) ± three doses extract (50, 120, and 300 mg tea polyphenols/kg body weight) q.d. for 4 weeks.	Induction of autophagy through increased Nrf2 activation and decreased Keap1 expression.	Dose-dependent improvement of functional and histopathological changes in hepatocytes after ethanol intake.
HEPFYGNEGALR (P03) [83]	Peptide isolated from *Apostichopus japonicus*.	Mice were given one intragastric dose of 50% ethanol (12 mL/kg) after oral P03 (20 mg/kg/d) or spermidine for 35 days and compared with controls without ethanol.	Induction of autophagy through the activation of the Nrf2/HO-1 pathway and blockade of NF-κB nuclear translocation.	Reduced hepatomegaly, liver inflammation, lipid droplet accumulation and increased antioxidant enzyme activities.
KD [84]	Major active ingredient extracted from *Anoectochilus roxburghii*.	Lieber–DeCarli mouse model ± 5% carbon tetrachloride in olive oil (intraperitoneal injection) and KD (20 40 mg/kg) or silymarin (80 mg/kg); control without ethanol.	Autophagy induction through AMPK activation.	KD alleviated alcoholic liver damage by reducing oxidative stress and lipid accumulation.
Lanthanum nitrate [85]	Rare earth element.	Acute ethanol intake mouse model (50%, 12 mL/kg, intragastric) after lanthanum nitrate (0.1, 0.2, 1.0, 2.0, and 20.0 mg/kg) administration for 30 days.	Induction of autophagy through the activation of the Keap1/Nrf2/p62 pathway.	Improved redox homeostasis and histopathological changes.
Melatonin [86]	Pineal gland hormone.	Acute ethanol intake mouse model (0.75 g/kg, intraperitoneal) ± melatonin (10 mg/kg, intraperitoneal) for 10 days.	Improved mitochondrial oxidation of NADH and decreased mitochondrial ability to oxidize FAD.	Prevented lysosomal destruction of liver tissue by limiting the increased activity of lysosomal enzymes and the resulting oxidative stress.
NAC [38]	Amino acid modified from L-cysteine.	Acute ethanol intake mouse model. Murine hepatocytes were exposed to ethanol ± NAC.	Reduction of autophagy via mTOR activation and reversed ROS levels.	NAC reduced TG and TBARS contents and ROS stress and reversed ethanol-induced mTOR inhibition.
PLT [87]	Protoberberine alkaloid.	Mouse hepatocytes were exposed to 75% alcohol for 2–3 weeks and PLT (0, 20, 50, 100, 150 and 200 μg/mL).	Induction of autophagy via AMPK/mTOR pathway activation.	PLT reduced ethanol-induced liver cell damage by inhibiting hepatocyte apoptosis through autophagy promotion.
PCP [88]	Gallic acid, lutein, quercetin, luteolin, apigenin, among others.	Acute ethanol intake (350 mM for 32 h) and/or PCP (100, 50 and 25 μg/mL) with zebrafish larvae.	Induction of autophagy through activating the AMPK/p62/Nrf2/mTOR signaling pathways and reduced oxidative stress.	PCP ameliorated ethanol-induced liver function damage and fat accumulation.
Procyanidin [89]	Polyphenol flavonoid.	Acute ethanol intake mouse model ± procyanidin (50 mg/kg for 11 days).	Induction of autophagy through increased LC3-II and reduced p62 levels, reduced ROS levels, and elevated GSH content.	Procyanidin eliminated lipid droplets and damaged mitochondria, thereby reducing hepatic lipid deposition and ROS overproduction.
Quercetin [90,91,92,93,94,95]	Plant-derived flavonoid.	Chronic and chronic plus single binge ethanol mouse models ± quercetin (control group without ethanol), L02 cells exposed to 3% ethanol for 24 h plus quercetin (20, 40, and 80 μM) for 24 h (control group without ethanol); transgenic zebrafish larvae were given quercetin (25, 50, and 100 μM for 48 h 3 days after fertilization) and ethanol for 32 h.	Induction of autophagy through FOXO3a activation and reversal of ethanol’s effects on AMPK and ERK2.	Quercetin inhibited inflammation and alleviated chronic ethanol-induced hepatic mitochondrial damage via mitophagy activation.
Rapamycin (sirolimus) [15,40,47]	Selective immunosuppressant (mTOR inhibitor).	Chronic and acute ethanol intake mouse models ± rapamycin.	Induction of autophagy via inhibition of mTOR signaling.	Rapamycin reduced ethanol-induced steatosis.
Resveratrol [46,96,97]	Dietary polyphenol.	Lieber–DeCarli mouse model plus acute ethanol binge, HepG2 cells exposed to oleic acid and alcohol.	Induction of autophagy via increased sirtuin-1 signaling.	Resveratrol increased the number of autophagosomes, reduced hepatic lipid accumulation, and protected against alcoholic liver steatosis.
SaIA [98]	Phenolic carboxylic acid extracted from *Salvia miltiorrhiza*.	Lieber–DeCarli mouse model with intragastric SaIA (8 and 16 mg/kg/d); AML-12 hepatocytes were examined.	Induction of autophagy via SIRT1 activation.	SaIA restored autophagosome-lysosome fusion, protected the liver from chronic ethanol exposure, decreased transaminase levels, attenuated histopathological liver damage, and prevented ethanol-induced liver cell damage in vitro.
Se-SP [99]	Microalga of the cyanobacterial class with chemical element enrichment.	Chronic ethanol intake mouse model (30%, 10 mL/kg by gavage for 15 days) and intragastric Se-SP (100, 200, and 400 mg/kg/d for 42 days).	Reduction of autophagy via decreased LC3 and increased p70s6k expression, and decreased p53, caspase 1, and 3 expression.	Se-SP protected against alcoholic liver damage by increasing antioxidant enzyme levels, inhibiting DNA damage and apoptosis, and inducing pyrosis.
Silibinin [100]	Flavonoid glycoside.	HepG2 and HL7702 cells exposed to ethanol or acetaldehyde and silibinin.	Induction of autophagy via PINK1 and Parkin activation.	Silibinin inhibited ethanol-induced ferroptosis, resolved oxidative stress, and reduced iron levels.
Simvastatin [101]	Statin.	Chronic ethanol intake rat model ± simvastatin (10 mg/kg/d).	Induction os autophagy, selectively inhibited HMG-CoA reductase.	Simvastatin ameliorated alcohol-induced liver histopathological changes, transaminase elevation, attenuated oxidative stress, and inflammation.
Sulforaphane [102]	Isothiocyanate derived from glucoraphanin present in *Brassica*.	Acute binge drinking mouse model ± sulforaphane (0.05 g/kg for 5 days), HepG2 (E47) cells treated with or without 100 mM ethanol ± 6 uM sulforaphane.	Induction of autophagy via Nrf2 activation.	Sulforaphane prevented binge ethanol–induced oxidative stress and steatosis in CYP2E1 KI mice and lipid accumulation in HepG2 (E47) cells.
Tangeretin [103]	Flavonoid derived from citrus peel.	Chronic binge drinking mouse model ± tangeretin (20 and 40 mg/kg).	Induction of autophagy via AMPK/Ulk1 signalling pathway activation.	Tangeretin dose-dependently normalized serum ALT and AST levels, liver weight, and serum and liver triacylglycerol contents; restored mitochondrial respiratory function; and suppressed steatosis.
TMP [104]	Alkylpyrazine extracted from *Ligusticum wallichii*.	Chronic ethanol intake mouse model ± TMP, LO2 cells exposed to ethanol (100 mM) and/or TMP (40 μM for 24 h).	Induction of autophagy via increased UQCRC2 expression and RIPK1/RIPK3 necrosome activation.	Reduced necroptosis and leakage of damage-associated molecular patterns and promoted the clearance of impaired mitochondria.
Torin 1 [55,105]	Pyridoquinoline (ATP-competitive mTOR kinase inhibitor).	Chronic plus binge ethanol intake mouse model ± torin 1.	Induction of autophagy via inhibition of mTORC1 and mTORC2 and increased hepatic TFEB levels.	Torin 1 reduced steatosis and liver damage induced by ethanol.
UDCA [106]	Hydrophilic bile acid (non–FXR agonistic).	Lieber–DeCarli mouse model ± UDCA.	Attenuated NF-κB activation.	UDCA attenuated and prevented the progression of alcoholic hepatic cholestasis.
Zinc (Zn) [107]	Chemical element.	VL-17A cells exposed to 100 mM ethanol for 24 h and 0, 10, 20, and 40 μM Zn for 48 h.	Induction of autophagy via ERK1/2 activation.	Zn depletion significantly suppressed autophagy, Zn exposure stimulated autophagy, cotreatment with ethanol, and 40 μM Zn had an additive effect on autophagy induction.

AT, Acer tegmentosum Maxim; HO-1, heme oxygenase 1; ACE, Antrodia cinnamomea extract; BBD, Babao Dan; BSE, barley sprout extract; ATG16L1, autophagy-related 16-like 1; CBS, cannabidiol; CBZ, carbamazepine; CMZ, chlormethiazole; PI3K, phosphoinositide 3 kinase; GSK3β, glycogen synthase kinase 3β; DMY, dihydromyricetin; TAX, dihydroquercetin; Sphk1, sphingosine kinase 1; SLC7A11, solute carrier family 7 member 11; GMC, glycycoumarin; HEPFYGNEGALR, histidine–glutamic acid–proline–phenylalanine–tyrosine–glycine–asparagine–glutamic acid–glycine–alanine–leucine–arginine; KD, kinsenoside; NAC, N-acetylcysteine; PLT, palmatine; PCP, Penthorum chinense Pursh; ERK2, extracellular signal–related kinase 2; SalA, salvianolic acid A; SeSP, selenium-enriched Spirulina platensis; HMG-CoA, 3-hydroxy-3- methylglutaryl-coenzyme A; Ulk1, uncoordinated-51-like kinase 1; TMP, tetramethylpyrazine; RIPK1/3, receptor-interacting serine/threonine-protein kinase 1/3; UDCA, ursodeoxycholic acid; Zn, zinc.

## Data Availability

No new data were created or analyzed in this study. Data sharing is not applicable to this article.
